# Isoniazid-Induced Acute Psychosis in a Patient with Pleural Tuberculosis

**DOI:** 10.1155/2019/4272941

**Published:** 2019-02-13

**Authors:** Joana Gomes, Diana Durães, André Sousa, Hugo Afonso

**Affiliations:** ^1^Department of Psychiatry and Mental Health, Centro Hospitalar Barreiro-Montijo, Avenida Movimento das Forças Armadas, 2834-003 Barreiro, Setúbal, Portugal; ^2^Department of Psychiatry and Mental Health, Hospital São Bernardo, Rua Camilo Castelo Branco, 2910-446 Setúbal, Portugal

## Abstract

Tuberculosis is one of the top 10 causes of death and the leading cause from a single infectious agent (above HIV/AIDS). Isoniazid is highly bactericidal against replicating tubercle bacilli and is a component of all antituberculous chemotherapeutic regimens currently recommended by the World Health Organization (WHO). Several neuropsychiatric adverse effects, following both therapeutic and overdose use of isoniazid, have been described and isoniazid-induced psychosis, although uncommon, has been reported in the literature. We describe the case of a 21-year-old black woman, with no prior psychiatric history, who developed a psychotic episode four days after she was started on isoniazid. This case highlights psychosis arising as a possible adverse effect of isoniazid and the importance of remaining vigilant when antituberculous therapy is started.

## 1. Introduction

Tuberculosis is one of the top 10 causes of death and the leading cause from a single infectious agent (above HIV/AIDS). In 2017, tuberculosis caused an estimated 1.3 million deaths (range, 1.2–1.4 million) among HIV-negative people and there were an additional 300 000 deaths from tuberculosis (range, 266 000–335 000) among HIV-positive people [1]. Effective drug treatments were first developed in the 1940s. The currently recommended treatment for cases of drug-susceptible tuberculosis is a six-month regimen of four first-line drugs: isoniazid, rifampicin, ethambutol, and pyrazinamide [1, 2]. Isoniazid, as part of this regimen, is a very commonly used drug and is also considered first-line treatment in latent tuberculosis [3].

Adverse effects of isoniazid, following both therapeutic and overdose use, have been reported. Psychosis associated with therapeutic isoniazid is a very dramatic, although infrequent adverse effect and its actual incidence rate is not well established. Some of the adverse effects of tuberculosis treatment are quite rare, and the relative infrequency of these adverse effects may explain the lack of either comprehensive randomized trials or epidemiological studies specifically targeting these adverse effects [4].

In this article, we report a case of a woman who developed a psychotic episode induced by isoniazid.

## 2. Case Presentation

A 21-year-old black woman, with no prior psychiatric history, presented at the Emergency Department of our hospital with an acute onset of psychotic symptoms.

These symptoms included paranoid delusion (she was convinced that her sister had made witchcraft against her and her boyfriend was cheating on her), psychomotor agitation, and initial insomnia.

The symptoms appeared four days after she was started on antituberculous therapy including isoniazid 300 mg/day, rifampicin 600 mg/day, ethambutol 1200 mg/day, and pyrazinamide 1500 mg/day, for pleural tuberculosis. She was also on pyridoxine 200 mg/day and thiamine 100 mg/day for prophylaxis against neuropathy associated with isoniazid.

In addition to the recently diagnosed pleural tuberculosis, the patient had no previous medical history and no history of substance abuse.

At mental state examination, she was poorly cooperative and suspicious, displayed psychomotor agitation, moving around constantly and had anxious humour and paranoid delusions. No errors of perception were detected and judgement regarding the morbid nature of her condition was impaired.

On examination, vital signs were stable and the physical signs, including neurological examination, were unremarkable. Testing including a complete blood count, chemistry panel, liver and thyroid function tests, and a urine toxicology screen was normal. A computed tomography scan of the head was obtained and showed no abnormality.

An initial diagnosis of drug-induced psychosis was made, once we considered the possibility that her psychotic symptoms could have been secondary to isoniazid, and the patient was admitted to our inpatient unit. All antituberculous therapy was discontinued and she was started on olanzapine 15mg/day.

By the seventh day, the psychotic symptoms had remitted, and the patient presented full insight into her clinical condition. The antituberculous therapy was reintroduced by the following order: rifampicin 600 mg/day at day 10, pyrazinamide 1500 mg/day at day 12, and ethambutol 1200 mg/day at day 17. Since these three antibacterial agents have efficacy in the treatment of pleural tuberculosis, it was decided not to introduce isoniazid.

At day 10, the antipsychotic started to be progressively reduced and by the time the patient was discharged (after 21 days of hospitalization) she was only taking olanzapine 5 mg/day and the antituberculous therapy.

The patient stopped taking olanzapine one week after discharge and at the four-week follow-up in outpatient consultation; she remained stable, with no recurrence of psychotic symptoms. The fast remission of symptoms and the good clinical outcome further supported our diagnosis of drug-induced psychosis.

## 3. Discussion

Tuberculosis accounts for millions of active disease cases and deaths in both developed and developing countries and although tuberculosis most commonly affects the lungs, any organ or tissue can be involved. In countries with comprehensive diagnostic and reporting systems, extrapulmonary tuberculosis (EPTB) accounts for 20–25% of reported cases. Of specific forms of EPTB, lymphatic, pleural, and bone or joint disease are the most common. Pulmonary and extrapulmonary disease should be treated with the same regimens [2]. In the presented case, our patient was diagnosed with pleural tuberculosis and initially treated with a regimen of four first-line drugs: isoniazid, rifampicin, ethambutol, and pyrazinamide.

Isoniazid [5–13], ethambutol [14, 15], and rifampicin [16] have all been documented to be associated with psychosis. In our patient, the temporal relationship between the administration of antituberculous therapy and the onset of psychotic symptoms, in the absence of prior psychiatric history, provided strong support for a diagnosis of drug-induced psychosis. Because most of the literature concerning this effect involves isoniazid, this was the agent we first consider as the most likely responsible for the psychotic episode. There is also evidence that patients who develop psychotic symptoms on one antituberculous may be at risk of further episodes secondary to another antituberculous agent [15]. Thus, rifampicin, pyrazinamide, and ethambutol were subsequently reintroduced, one by one, during the hospitalization period to assure close monitoring of any possible arising symptoms.

Isoniazid, the hydrazide of isonicotinic acid, is highly bactericidal against replicating tubercle bacilli and is a component of all antituberculous chemotherapeutic regimens currently recommended by WHO [2, 3]. Isoniazid is generally well tolerated at recommended doses [2]. The most common adverse effects are those of the liver, manifested most often by an asymptomatic rise in serum concentrations of hepatic transaminases, followed by neuropsychiatric effects. These neuropsychiatric effects include perceived cognitive impairment and lethargy (the most common), headaches, blurred vision, peripheral neuropathy, sleep disturbance, and depression. Other uncommon neuropsychiatric disturbances including optic neuritis, generalized convulsions, and psychosis can develop in susceptible individuals [2, 17].

Isoniazid-induced psychosis, although infrequent, has been reported in patients with [6, 7] and without a psychiatric history [8–14, 18], both in isoniazid monotherapy or in combination with other antituberculostatic drugs. The onset of psychotic symptoms after initiation of therapy varies considerably, ranging from days to months and these symptoms included paranoid delusions, auditory, visual and tactile hallucinations, suicidality, echolalia, and psychomotor agitation [6–14, 18, 19]. Our patient presented with psychomotor agitation, anxious humour and paranoid delusions arising four days after initiating treatment.

The mechanism of isoniazid-induced psychosis is not well understood, but isoniazid is known to interfere with several metabolic pathways essential for normal neuronal functioning. Pallone et al. suggested two hypotheses regarding the mechanism of isoniazid-associated psychosis [20]. The first is that isoniazid acts as a monoamine oxidase (MAO) inhibitor, preventing the degradation of catecholamines and serotonin with a resultant increase in the concentrations of these neurotransmitters within the Central Nervous System (CNS). The other mechanism proposed by Pallone et al. entails pyridoxine deficiency induced by isoniazid. Isoniazid may lead to pyridoxine deficiency since it combines with pyridoxal and this complex inhibits the activity of pyridoxal kinase. The result is a disturbance in the normal tryptophan metabolism. Since pyridoxine deficiency may be secondary to the prescription of isoniazid, pyridoxine supplementation has been thought to prevent this situation. Nevertheless, the role of pyridoxine supplementation in prevention or treatment of isoniazid-associated psychiatric symptoms is unclear since the systematic use of pyridoxine does not seem to prevent the emerging of psychosis, suggesting that pyridoxine deficiency could only have a partial implication [8, 13]. It was proposed that higher doses of pyridoxine (e.g., 50 mg/day) would be probably required to prevent the occurrence of isoniazid-induced psychosis [13]. In the presented case, pyridoxine 200mg/day did not prevent isoniazid-induced psychosis.

Additionally, it is also claimed that isoniazid may cause oxidative stress, which can cause decreased levels of N-metil-D-aspartate (NMDA) 2A receptors in the hippocampus [21]. Changes in NMDA receptor density may be one of the mechanisms of isoniazid-induced neuropsychiatric disorders.

Isoniazid is metabolized in the body mainly to its acetyl derivatives. Patients can be broadly classified into two groups: the slow acetylators and the rapid acetylators [22]. The slow acetylators are at risk for drug accumulation and consequently more side effects [12]. A previous personal history of psychiatric or neurological disorder, alcoholism, diabetes mellitus, malnutrition, uraemia, hepatic insufficiency, and a dose of isoniazid above 5mg/kg have all been described as predisposing factors for isoniazid-induced psychosis [6, 12, 20, 23].

Treatment for isoniazid-induced psychosis includes discontinuation of isoniazid [10, 12, 13], addition of an antipsychotic [6, 7], or a combination of both [5, 9, 11]. Given the psychomotor agitation and insomnia presented by our patient, we choose to introduce olanzapine for its sedative properties, along with the discontinuation of isoniazid.

## 4. Conclusion

The reported case highlights psychosis arising as an infrequent adverse effect of isoniazid, emphasising the need for the physicians to be aware about the toxicity profiles of antitubercular drugs. Acute onset of psychotic symptoms in a patient taking isoniazid should lead to suspicion of this psychiatric side effect and to a prompt intervention, involving the discontinuation of isoniazid and/or a trial of an antipsychotic. The use of pyridoxine in prevention or treatment of isoniazid-induced psychosis remains controversial, as exemplified by our case, and needs to be further studied.

As protective measures the authors suggest tailoring isoniazid dose to weight, eventual genetic testing if slow acetylation is suspected, and close monitoring of patients with the previous mentioned risk factors.
